# A nomogram model based on albumin-bilirubin score for predicting 90-day prognosis in patients with acute-on-chronic liver failure

**DOI:** 10.3389/fmed.2024.1406275

**Published:** 2025-01-06

**Authors:** Wei Ding, Jiandong Shen, Li Zhang, Jianguo Shao, Zhaolian Bian, Hong Xue

**Affiliations:** ^1^Medical School of Nantong University, Nantong, Jiangsu, China; ^2^Nantong Third People’s Hospital, Affiliated Nantong Hospital 3 of Nantong University, Nantong, Jiangsu, China

**Keywords:** acute-on-chronic liver failure, prognosis, nomogram, risk factor, ascites

## Abstract

**Objective:**

To develop a nomogram model based on the albumin-bilirubin (ALBI) score for predicting the 90-day prognosis of patients with acute-on-chronic liver failure (ACLF) and to evaluate its predictive efficacy.

**Methods:**

Clinical data of 290 ACLF patients at the Third People’s Hospital of Nantong City, collected from December 2020 to December 2023, were analyzed. The data were divided into a training set (*n* = 200) and a validation set (*n* = 90), with August 2022 as the cut-off date. Patients in the training set were categorized into an improvement group (*n* = 133) and a mortality group (*n* = 67) based on their 90-day outcomes. The predictive power of baseline parameters was assessed using univariate and multivariate logistic regression to construct model. Model performance was assessed using receiver operating characteristic (ROC) curves, calibration curves, decision curve analysis (DCA) and the Hosmer-Lemeshow test.

**Results:**

Creatinine (CR) [odds ratio (OR) = 1.013, 95% confidence interval (CI): 1.004–1.022], ALBI (OR = 10.831, 95% CI: 4.009–33.247), Gender (OR = 1.931, 95% CI: 0.973–3.870) and ascites (OR = 3.032, 95% CI: 1.249–8.178) were identified as independent prognostic factors. The prognostic model formula was derived as prognostic index (PI) = –0.591 + 0.658 × Gender + 1.109 × ascites + 0.012 × CR + 2.382 × ALBI. The area under the curve (AUC) was 0.804 (95% CI: 0.741–0.866), with a specificity of 85.0% and a sensitivity of 65.7% at a cut-off of 0.425. The AUC of the validation set was 0.811 (95% CI: 0.697–0.926). The Hosmer-Lemeshow test indicated a good model fit with a *p*-value of 0.287 for the training set and 0.423 for the validation set. Calibration curves demonstrated the accuracy of the model, and DCA results suggested that the model was clinically useful when the threshold was below 0.6.

**Conclusion:**

The nomogram model incorporating ALBI with CR, Gender and ascites can predict the 90-day prognosis of ACLF patients, potentially helping to optimize treatment strategies and improve patient outcomes.

## Introduction

Acute-on-chronic liver failure (ACLF) is a syndrome that occurs in the context of chronic liver disease and is triggered by a variety of internal and external liver insults (1). Characterized by acute decompensation, multi-organ failure and high short-term mortality, ACLF poses a significant challenge to healthcare systems worldwide, particularly in Asia where liver disease is prevalent (2, 3). Patients with ACLF often present with complications such as hepatorenal syndrome, ascites, hepatic encephalopathy and bacterial infections, which further complicate their clinical course.

The pathogenesis of ACLF is multifaceted, involving immune responses, hepatic inflammation and gut microbiome dysbiosis, among other factors. Early diagnosis of ACLF can be challenging due to overlapping symptoms and signs with other liver diseases (3). Liver transplantation remains the only curative option for ACLF; however, the shortage of donor organs limits its widespread use. Therefore, early diagnosis, prompt treatment and comprehensive management are essential to improve patient outcomes and survival.

Several prognostic models for ACLF have been developed, including the APASL-AARC score, EASL-CLIF C ACLF score, COSSH-ACLF II score, Model for End-Stage Liver Disease (MELD) and MELD-Na scores (1). These models provide clinicians with objective criteria to assess the severity of ACLF and facilitate informed treatment decisions and resource allocation. The Albumin-Bilirubin (ALBI) score, originally proposed by Johnson et al. as a model to assess liver function in patients with hepatocellular carcinoma (HCC), has shown limited predictive power for the prognosis of ACLF (4).

Our aim is to use existing case data to develop and validate a novel ALBI prognostic model and nomogram to predict the prognosis of ACLF patients. Nomograms offer a more intuitive and user-friendly approach compared to statistical models, helping clinicians to make more accurate medical decisions. By integrating ALBI with other clinically relevant parameters, we aim to improve the prognostic accuracy and clinical utility of this model, ultimately contributing to improved treatment strategies and patient outcomes in the management of ACLF.

## Study design and participants

### Study participants

The diagnostic criteria for acute-on-chronic liver failure (ACLF) are taken from the “Acute-on-chronic liver failure: Consensus recommendations of the Asian Pacific association for the study of the liver (APASL).” The details are as follows the patient presents with acute liver injury characterized by jaundice [serum bilirubin ≥5 mg/dL (85 μmol/L)] and coagulopathy [International Normalized Ratio (INR) ≥1.5 or prothrombin activity <40%] complicated by clinical ascites and/or hepatic encephalopathy within 4 weeks, in the context of previously diagnosed or undiagnosed chronic liver disease/cirrhosis (5). Exclusion criteria are as follows (1) A history of liver transplantation prior to the study. (2) Pregnancy or lactation in women. (3) Coexistence of malignant tumors. (4) Age less than 18 years. (5) Hospitalization of less than 3 days. (6) Diseases such as choledocholithiasis and hemolytic disorders can lead to elevated bilirubin levels. This study is a retrospective cohort analysis conducted in the Third People’s Hospital of Nantong City. The research was approved by the Ethics Committee of the Third People’s Hospital of Nantong City with protocol number EK2023027. All procedures were performed in accordance with relevant guidelines and regulations. Clinical data of 290 patients diagnosed with ACLF between December 2020 and December 2023 were collected and divided into two groups: a training set (*n* = 200) including patients from December 2020 to August 2022, and a validation set (*n* = 90) including patients from September 2022 to December 2023 (Figure 3).

Patient demographics, etiology of cirrhosis (alcoholic liver disease, cholestatic liver disease, other) and complications (ascites, hepatic encephalopathy, hepatorenal syndrome, bacterial infections, gastrointestinal bleeding) were obtained from the hospital information system. Laboratory parameters included alanine aminotransferase (ALT), aspartate aminotransferase (AST), total bilirubin (TBIL), albumin (ALB), gamma glutamyl transferase (GGT), alkaline phosphatase (ALP), international normalized ratio (INR), prothrombin time activity (PTA), white blood cell count (WBC), neutrophil/lymphocyte ratio (NLR), creatinine (CR) and sodium (Na). The ALBI score was calculated as ALBI = (log_10_ (TBIL)) × 0.66 – (ALB × 0.085), with TBIL in μmol/L and ALB in g/L. The MELD score was determined using the formula 3.78 × ln(TBIL) + 11.2 × ln(INR) + 9.57 × ln(CR) + 6.43 × (aetiological_factor_coefficient) (6), with a coefficient of 0 for cholestatic or alcoholic liver disease and 1 for other aetiologies. MELD-Na was calculated as MELD + 1.59 × (135 – serum_sodium_concentration), with serum sodium in mmol/L (7, 8).

### Statistical analysis

Data were processed using R-4.3.2 and SPSS 27.0 statistical software. R packages used include forestplot, officer, flextable, shiny, shinydashboard, shinyWidgets, dplyr, pROC, car, rms, pROC, Hmisc, rmda. The Kolmogorov–Smirnov (K-S) test was used to assess the normality of the data set. Continuous variables with normal distribution were expressed as mean ± standard deviation (SD) and compared between groups using independent samples *t*-tests; non-normally distributed continuous variables were expressed as median (P25, P75) and compared using Mann–Whitney *U* tests (9); categorical variables were expressed as frequencies and analyzed using chi-squared tests. Significant baseline data were analyzed using single and multivariable logistic regression to construct the model and generate a nomogram for predicting 90-day prognosis after ACLF diagnosis. Internal validation was performed using the validation set (*n* = 90) to assess the predictive performance and discriminative ability of the final model, as measured by the area under the receiver operating characteristic (ROC) curve (AUC) (10–12). Calibration was verified using calibration curves, goodness of fit was assessed using the Hosmer-Lemeshow test (13), and clinical utility was assessed using decision curve analysis (DCA) (14). In multivariable analysis, a *p* value <0.1 was considered statistically significant for trend, and a *p* value <0.05 was considered statistically significant for other comparisons (15, 16).

## Results

### Patient characteristics

Between December 2020 and December 2023, a total of 290 patients with ACLF were treated at the Third People’s Hospital of Nantong City and were categorized into a training set and a validation set. The general characteristics of patients in both cohorts were analyzed, and no statistically significant differences were observed in terms of age, hepatic encephalopathy, bacterial infections, gastrointestinal bleeding, hepatorenal syndrome, ALT, AST, ALB, GGT, ALP, INR, PTA, WBC, NLR, CR, Na, MELD score, MELD-Na score, and ALBI score (*p* > 0.05) (Table 1). Significant differences were found in the presence of ascites, gender distribution, and total bilirubin (TBIL) levels (*p* < 0.05).

**Table 1:** Baseline characteristics of the training and validation cohorts.

Parameter	Training set (*n* = 200)	Validation set (*n* = 90)	*χ*^2^/*t*/*Z* value	*p* value
Age [years, M (P25, P75)]	57.00 (50.00, 66.00)	55.00 (47.75, 71.00)	−0.283	0.777
Gender (Male/Female, *n*)	127/73	72/18	7.848	0.005
Ascites (Yes/No)	156/44	53/37	11.262	0.001
Hepatic encephalopathy (Yes/No)	43/157	17/73	0.258	0.612
Bacterial infections (Yes/No)	182/18	85/5	1.008	0.315
Gastrointestinal bleeding (Yes/No)	29/171	9/81	1.104	0.293
Hepatorenal syndrome (Yes/No)	8/192	1/89	1.723	0.189
Hepatitis B, *n* (%)	156 (78.00)	69 (76.67)	0.063	0.801
Hepatitis C, *n* (%)	15 (7.50)	10 (11.11)	1.027	0.311
Alcoholic liver disease, *n* (%)	21 (10.50)	5 (5.55)	1.859	0.173
Other liver disease, *n* (%)	8 (4.00)	6 (6.67)	0.961	0.327
ALT [U·L^−1^, M (P25, P75)]	136.50 (42.75, 652.75)	196.00 (64.00, 831.75)	−1.464	0.143
AST [U·L^−1^, M (P25, P75)]	145.00 (74.25, 536.25)	203.00 (73.00, 691.25)	−1.019	0.308
TBIL [μmol·L^-1, M (P25, P75)]	265.75 (166.10, 377.32)	230.95 (97.90, 346.70)	−2.178	0.029
ALB [g·L^−1^, M (P25, P75)]	30.45 (27.62, 33.10)	30.40 (27.00, 33.72)	−0.321	0.748
GGT [U·L^−1^, M (P25, P75)]	95.5 (58.75, 169.75)	112.00 (66.00, 195.50)	−1.029	0.303
ALP [U·L^−1^, M (P25, P75)]	149.50 (116.25, 209.75)	142.50 (103.00, 188.50)	−1.031	0.303
INR [M (P25, P75)]	1.72 (1.46, 2.07)	1.72 (1.46, 2.16)	−0.817	0.414
PTA (%) [M (P25, P75)]	32.04 (22.60, 41.77)	32.35 (21.57, 42.86)	−0.136	0.892
WBC [×10^9^/L, M (P25, P75)]	6.30 (4.23, 8.94)	6.43 (4.51, 9.58)	−0.667	0.504
NLR [M (P25, P75)]	4.10 (2.43, 7.30)	4.91 (2.73, 9.42)	−1.321	0.187
CR [μmol·L^−1^, M (P25, P75)]	65.00 (52.42, 83.00)	67.65 (56.90, 94.20)	−0.890	0.373
Na^+^ [mmol·L^−1^, M (P25, P75)]	135.60 (131.55, 138.50)	135.70 (132.50, 138.35)	−0.545	0.586
MELD score [points, M (P25, P75)]	18.16 (14.80, 22.89)	19.27 (14.75, 23.23)	−0.527	0.598
MELD-Na score [points, M (P25, P75)]	18.51 (11.58, 29.09)	17.47 (11.60, 24.93)	−0.515	0.607
ALBI score [points, M (P25, P75)]	−0.96 (−1.21, −0.70)	−1.07 (−1.37, −0.81)	1.348	0.0534

In the analysis of the data, the independent samples *t*-test was used to compare the means of continuous variables that followed a normal distribution. For continuous variables that did not follow a normal distribution, the Mann–Whitney *U* test was used to assess differences. Categorical variables were analyzed using the Chi-square test (*χ*2). A *p* value of less than 0.05 was considered statistically significant. These methods were chosen to ensure robust and appropriate statistical inference within the study.

The prognosis of patients in the training cohort diagnosed with ACLF was assessed at 90 days post-diagnosis and categorized into an improvement group and a mortality group. Comparative analysis of these groups revealed significant differences in several clinical and laboratory parameters. Age, gender, ascites, hepatic encephalopathy, bacterial infections, gastrointestinal bleeding, hepatorenal syndrome, TBIL, ALB, INR, PTA, WBC, NLR, CR, Na, MELD score, MELD-Na score, and ALBI score were identified as factors significantly associated with patient mortality (*p* < 0.05) (Tables 2, 3).

**Table 2:** Baseline characteristics of patients in the training cohort.

Variable	Improvement group (*n* = 133)	Mortality group (*n* = 67)	*χ*2/*t*/*Z* value	*p* value
Age (years, mean ± SD)	55.62 ± 12.34	60.27 ± 11.34	−2.584	0.010
Gender (Male/Female, *n*)	92/41	35/32	5.513	0.019
Ascites (Yes/No)	95/38	61/6	9.991	0.002
Hepatic encephalopathy (Yes/No)	16/117	27/40	21.096	<0.001
Bacterial infections (Yes/No)	116/17	66/1	6.934	0.008
Gastrointestinaln bleeding (Yes/No)	12/121	17/50	9.608	0.002
Hepatorenal syndrome (Yes/No)	1/132	7/60	10.908	0.001
Hepatitis B, *n* (%)	100 (75.19)	48 (71.64)	0.291	0.589
Hepatitis C, *n* (%)	12 (9.02)	7 (10.45)	0.105	0.746
Alcoholic liver disease, *n* (%)	8 (6.02)	5 (7.46)	0.154	0.695
Other liver disease, *n* (%)	13 (9.77)	7 (10.45)	0.022	0.881
ALT [U·L^−1^, Median (P25, P75)]	147.00 (47.50, 665.50)	76.00 (36.00, 583.00)	−1.598	0.110
AST [U·L^−1^, Median (P25, P75)]	153.00 (73.00, 542.00)	140.00 (80.00, 519.00)	−0.118	0.906
TBIL (μmol·L^−1^, mean ± SD)	253.79 ± 151.22	329.02 ± 134.04	−3.446	0.001
ALB [g·L^−1^, Median (P25, P75)]	31.10 (28.70, 33.30)	28.60 (25.30, 31.0)	−4.217	<0.001
GGT [U·L^−1^, Median (P25, P75)]	105.00 (65.00, 182.50)	91.00 (53.00, 149.00)	−1.032	0.302
ALP [U·L^−1^, Median (P25, P75)]	154.00 (117.00, 212.00)	143.00 (94.00, 196.00)	−0.821	0.412
INR [Median (P25, P75)]	1.59 (1.35, 1.91)	1.88 (1.65, 2.31)	−4.386	<0.001
PTA (%) [Median (P25, P75)]	33.00 (26.04, 51.17)	26.19 (19.30, 33.33)	−4.415	<0.001
WBC [×10^9^/L, Median (P25, P75)]	5.740 (4.04, 8.22)	7.26 (4.78, 10.29)	−2.392	0.017
NLR [Median (P25, P75)]	3.36 (2.30, 6.35)	5.49 (2.86, 10.41)	−3.023	0.003
CR [μmol·L^−1^, Median (P25, P75)]	62.60 (50.60, 73.80)	79.10 (59.00, 115.00)	−3.863	<0.001
Na^+ [mmol·L^−1^, Median (P25, P75)]	136.50 (132.75, 138.75)	133.0 (128.30, 137.20)	−3.127	0.002
MELD Score [points, Median (P25, P75)]	17.00 (13.85, 20.06)	22.81 (18.19, 26.45)	−5.851	<0.001
MELD-Na Score [points, Median (P25, P75)]	15.74 (9.09, 21.77)	26.33 (18.46, 34.50)	−5.315	<0.001
ALBI Score [points, Median (P25, P75)]	−1.05 (−1.33, −0.86)	−0.77 (−0.97, −0.53)	−5.274	<0.001

In the statistical analysis of the data, the Kolmogorov–Smirnov (K-S) test was used to assess the normality of the data set, differences in normally distributed continuous variables were compared using the independent samples t-test, while non-normally distributed continuous variables were assessed using the Mann–Whitney *U* test. Categorical variables were assessed using the chi-squared test (*χ*^2^). A threshold of *p* < 0.05 was set to indicate statistically significant differences. This approach ensured a rigorous and standardized method for assessing the statistical significance of the observed data across different parameters.

**Table 3:** Univariate and multifactorial logistic regression analysis of 90-day prognosis in ACLF patients.

Variable	Number of patients	Univariate logistic regression		Multivariate logistic regression	
		Odds ratio (95% CI)	*p*-value	Odds ratio (95% CI)	*p*-value
Age (years)	200	1.010 [0.985, 1.035]	0.441		
INR	200	1.133 [0.953, 1.682]	0.335		
PTA (%)	199	1.001 [0.993, 1.009]	0.764		
WBC (×10^9^/L)	200	0.985 [0.915, 1.053]	0.664		
NLR	199	1.008 [0.985, 1.033]	0.483		
CR (μmol·L^−1^)	200	1.013 [1.006, 1.022]	<0.001	1.013 [1.004, 1.022]	<0.001
ALBI (points)	200	11.359 [4.459, 32.534]	<0.001	10.831 [4.009，33.247]	<0.001
Gender					
Male	127				
Female	73	2.052 [1.122, 3.768]	0.025	1.931 [0.973, 3.870]	0.063
Ascites					
No	44				
Yes	156	2.302 [1.071, 5.405]	0.042	3.032 [1.249, 8.178]	0.021
Hepatic encephalopathy					
No	157				
Yes	43	1.082 [0.522, 2.177]	0.832		
Bacterial infections					
No	18				
Yes	182	2.712 [0.856, 12.020]	0.132		
Gastrointestinal bleeding					
No	171				
Yes	29	0.723 [0.286, 1.676]	0.475		
Hepatorenal syndrome					
No	192				
Yes	8	1.200 [0.240, 5.046]	0.817		

Key laboratory parameters such as International Normalized Ratio (INR), platelet count (PTA), white blood cell count (WBC), neutrophil-to-lymphocyte ratio (NLR), creatinine (CR) and albumin bilirubin (ALBI) score are meticulously analyzed in the context of prognostic assessment of patients with Acute Chronic Liver Failure (ACLF). A threshold of *p* < 0.1 is used to define statistical significance, ensuring that only the most robust predictors are included in the multivariate model.

### Univariate and multifactorial analysis of 90-day prognosis in ACLF

In the multivariate analysis, factors that showed statistical significance in the univariate setting (*p* < 0.05) were further evaluated to determine their independent association with 90-day prognosis in patients with ACLF. Due to clinical considerations and intercorrelations between variables, ALB, TBIL, Na ions, MELD score, MELD-Na score were not included in the univariate analysis. Instead, age, Gender, ascites, hepatic encephalopathy, bacterial infection, gastrointestinal bleeding, hepatorenal syndrome, INR, PTA, WBC, NLR, CR and ALBI score were selected for inclusion in the multivariate model. The results showed that CR [odds ratio (OR) = 1.013, 95% confidence interval (CI): 1.004, 1.022], ALBI (OR = 10.831, 95% CI: 4.009, 33.247), Gender (OR = 1.931, 95% CI: 0.973, 3.870) and ascites (OR = 3.032, 95% CI: 1.249, 8.178) emerged as significant independent predictors of patient prognosis (*p* < 0.1).

### The nomogram for predicting 90-day prognosis in patients with ACLF

**Figure 1:**
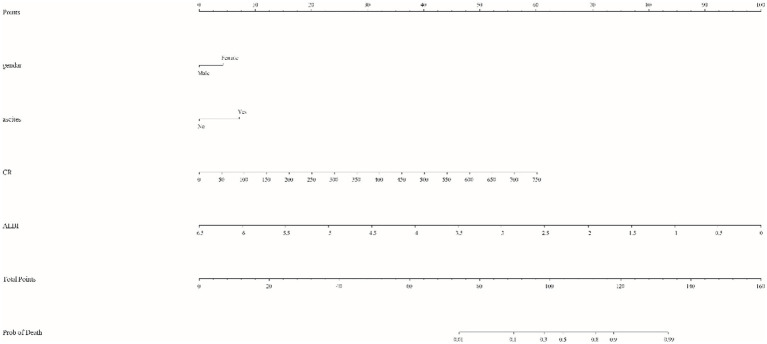
Nomogram for predicting 90-day mortality risk in patients with acute-on-chronic liver failure (ACLF). The model integrates critical clinical variables, including gender (female), ascites (present), creatinine (CR) and albumin bilirubin (ALBI) score. Each parameter is assigned a specific score based on the coefficients derived from multivariate logistic regression analysis, allowing for a personalized risk assessment by summing the assigned scores. The total score is then translated onto a scale to provide an estimated probability of mortality, thereby assisting clinicians in prognostic discussions and therapeutic strategy.

Taking into account clinical evidence and despite a *p* value exceeding the conventional threshold of 0.05 for gender, it was judiciously included in the model. Logistic regression analysis identified significant predictors - CR, ALBI, ascites and gender - which were included in the prognostic model with the equation: $\begin{array}{l} \mathrm{PI}=-0.591+0.658\times \mathrm{Gender}+1.109\times \mathrm{ascites}+0.012\times CR\\ +2.382\times \mathrm{ALBI} \end{array}$. This model facilitated the construction of a nomogram that allowed the calculation of a total score by summing the points assigned to Gender, ascites, creatinine and ALBI. The total score was then used to estimate risk (ranging from 0.01 to 0.99). For example, a female patient (5 points) with ultrasound-confirmed ascites (8 points), a creatinine level of 403 μmol/L (33 points) and an ALBI score of −1.85 (70 points) would accumulate a total score of 116, corresponding to a risk of 0.86. A higher total score indicates an increased likelihood of mortality within 90 days (Figure 1). This nomogram serves as a valuable tool for clinicians to communicate probabilistic outcomes and guide decision making in the management of patients with ACLF.

### Evaluate the effectiveness of the model

**Figure 2:**
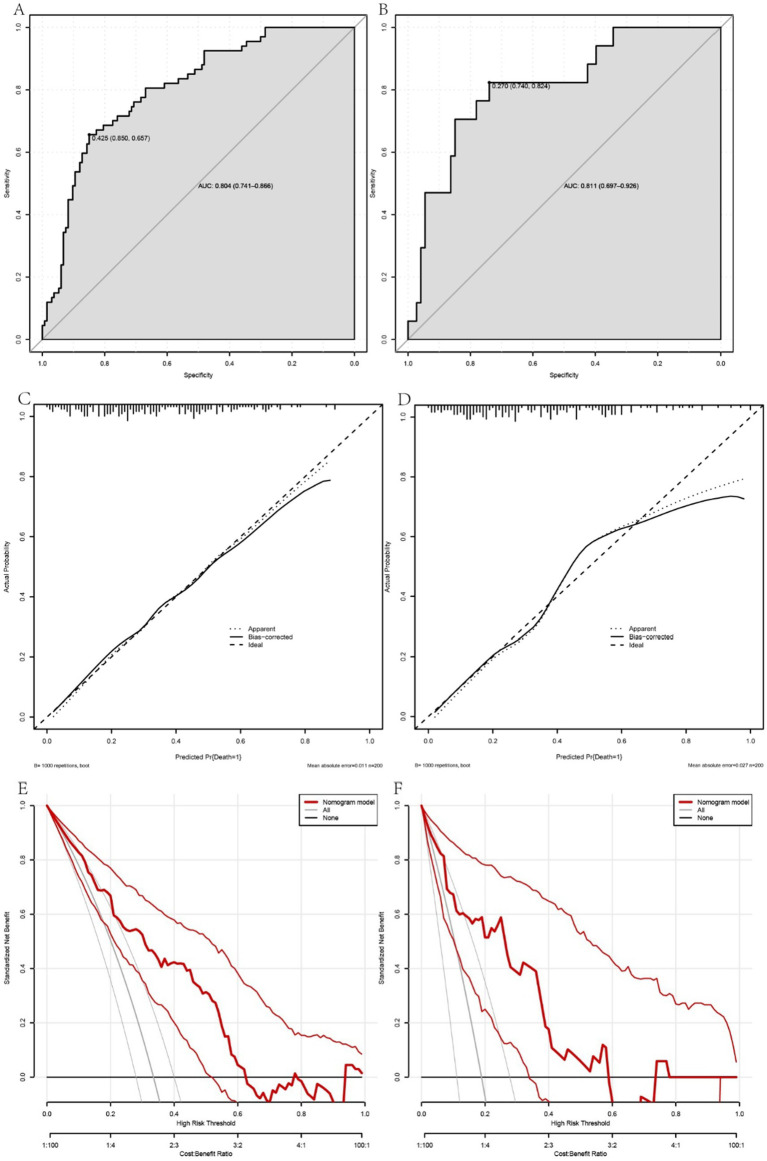
A comprehensive evaluation of the performance of the prognostic model in predicting the occurrence of 90-day mortality events in patients with acute-on-chronic liver failure (ACLF). Panels **A**, **C** and **E** show the receiver operating characteristic (ROC) curve, calibration plots and decision curve analysis (DCA) for the training cohort, while panels **B**, **D** and **F** correspond to the validation cohort. The ROC curves **(A,B)** illustrate the discriminative ability of the model, with the training set **(A)** showing an area under the curve (AUC) reflecting good predictive accuracy. The calibration plots **(C,D)** show the model’s consistency between observed and predicted probabilities across different risk strata, indicating a moderate level of agreement. The DCA **(E,F)** provides insight into the clinical utility of the model by comparing it with alternative strategies, highlighting the net benefit of the model at different threshold probabilities. Together, these analyses underscore the reliability and clinical applicability of the model, providing a valuable prognostic tool for the management of ACLF patients.

To assess the efficacy of the nomogram model for predicting 90-day mortality in patients with ACLF, a number of analytical techniques were used, including ROC curve construction, calibration plots and DCA. The predictive accuracy and discriminative ability of the model was reflected by the ROC curve, with the training set having an AUC of 0.804 (95% confidence interval [CI] = 0.741 to 0.866) using a cut-off value of 0.425, corresponding to a specificity of 85.0% and a sensitivity of 65.7% (Figure 2A). The validation set showed an AUC of 0.811 (95% CI = 0.697 to 0.926), indicating satisfactory predictive performance (Figure 2B).

Calibration plots for both the training and validation cohorts showed moderate agreement, with the accuracy of the model considered acceptable (Figures 2C,D). In addition, DCA, when combined across the training and validation sets, showed that the model’s curve outperformed the chance and ‘treat all’ or ‘treat none’ extremes when the threshold was below 0.6. This suggests that use of the nomogram for prognostication and subsequent clinical decision making in ACLF provides greater net benefit (Figures 2E,F). The Hosmer-Lemeshow test confirmed the good fit of the model, with a *p*-value of 0.287 for the training set and 0.423 for the validation set, highlighting the robust calibration of the model.

**Figure 3:**
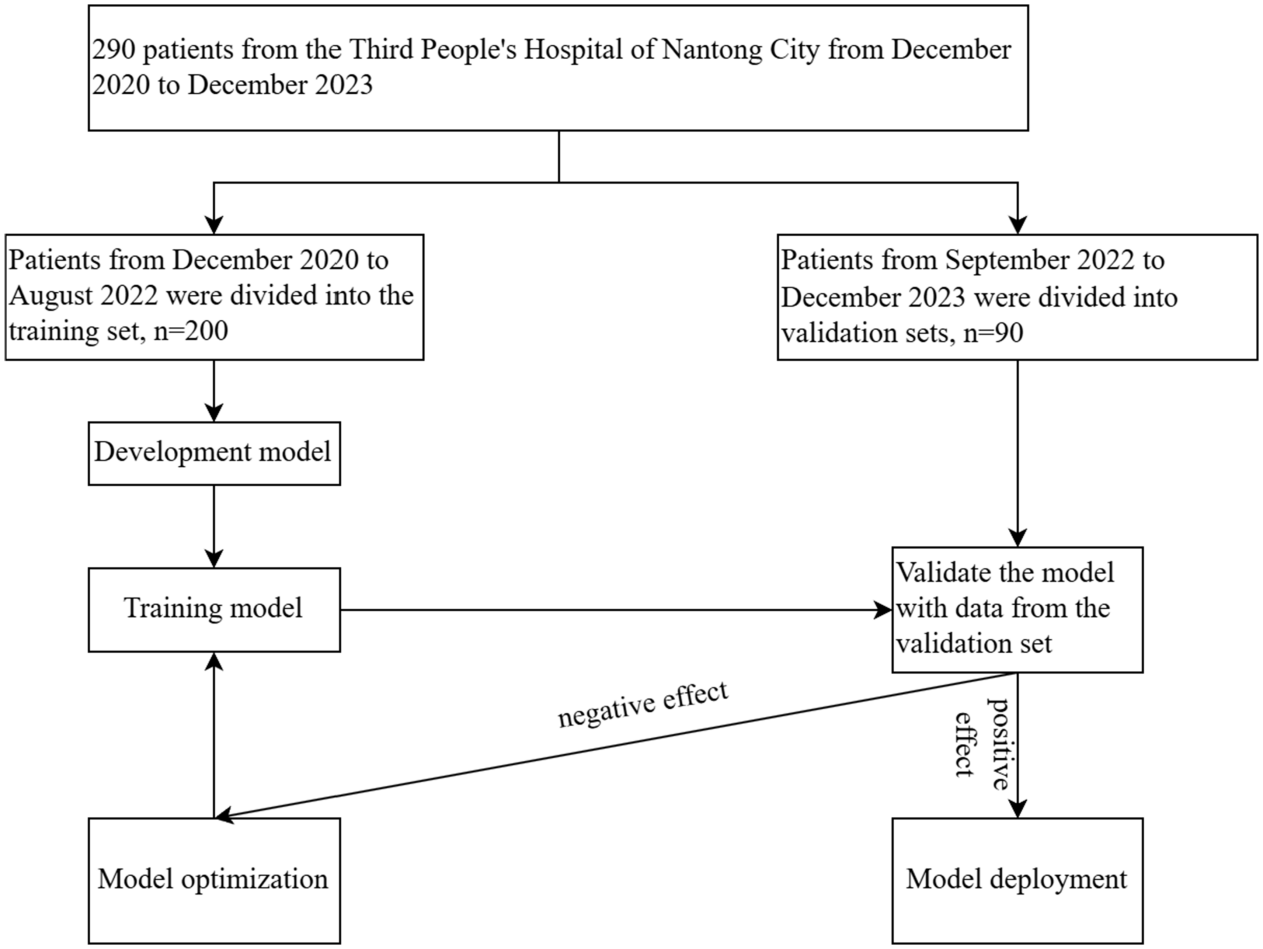
Clinical data of 290 ACLF patients at the Third People’s Hospital of Nantong City, collected from December 2020 to December 2023, were analyzed. The data were divided into a training set (*n* = 200) and a validation set (*n* = 90), with August 2022 as the cut-off date. The model was developed through training on the dataset, followed by performance validation using the validation set. Subsequent optimizations were conducted to refine the model, culminating in its final confirmation.

**Figure 4:**
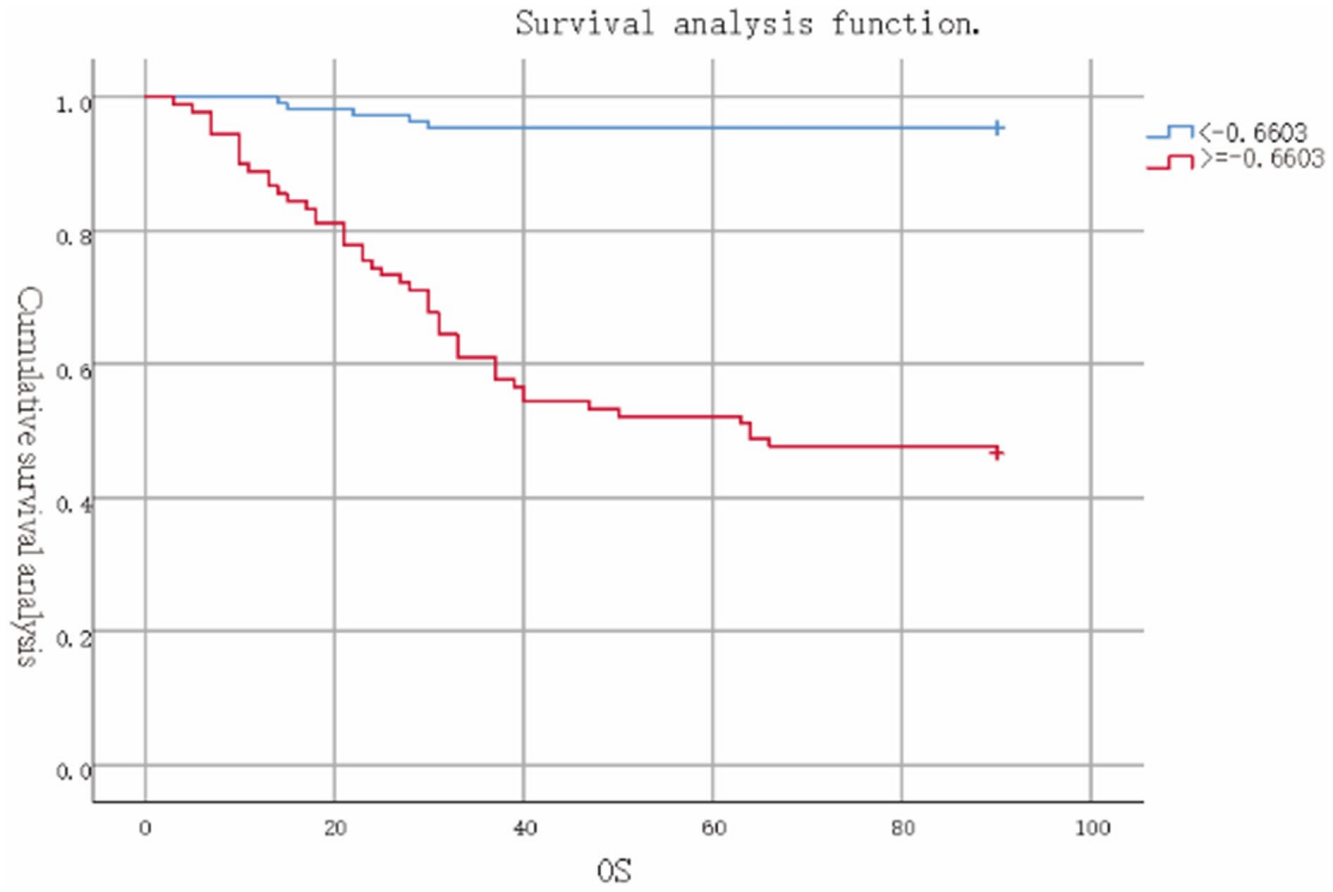
Survival analysis of the high model scoring group versus the low model scoring group.

These rigorous evaluations validate the nomogram as a reliable tool for prognostic stratification in ACLF, providing clinicians with a nuanced tool to assess mortality risk and inform patient management strategies (Figures 3, 4).

## Discussion

ACLF represents a critical transition in chronic liver disease, triggered by an acute insult, leading to rapid deterioration of liver function and potentially culminating in multi-organ failure within a short period of time (17, 18). Given the high mortality associated with ACLF and the paucity of effective therapeutic interventions, early diagnosis and prognostic assessment are of paramount importance in clinical practice (19). The development and evaluation of prognostic models is essential to improve our understanding of disease progression, refine treatment strategies and provide patients with more accurate prognostic information.

Despite the widespread use of the CTP score and the MELD score in liver disease, their predictive power in China is not optimistic due to the differences in aetiological factors and environmental conditions between regions (20–22). To address this gap, we have developed a survival prediction model that integrates the ALBI with CR, gender and ascites, complemented by a nomogram to facilitate clinical application. Our model has undergone rigorous internal validation and has demonstrated commendable efficacy.

The ALBI score, introduced by Professor Philip J. Johnson and colleagues (4), emerged from a landmark study in 2015 that analyzed clinical data from 1,313 patients with hepatocellular carcinoma (HCC) of various stages in Japan. By incorporating serum albumin and total bilirubin, the ALBI score was developed to measure liver reserve function. The score has been shown to be an effective predictor of overall survival in patients with mid-stage HCC. However, its efficacy in the prognosis of ACLF was suboptimal (23–25). In our study, the ALBI score was −1.05 (−1.33, −0.86) in the improvement group and − 0.77 (−0.97, −0.53) in the mortality group, with an AUC of 0.729, Our findings are in agreement with those of Bo Chen’s study (26), which evaluated the relative efficacy of the ALBI score in predicting the 90-day mortality rate of patients with acute-on-chronic liver failure (ACLF) by establishing a ROC curve. The area under the ALBI score curve (AUC) was 0.784 ± 0.049 (*p* < 0.001), indicating that they also highlighted the significance of the ALBI score in predicting the exacerbation of chronic liver failure, similar to the AUC of 0.729 observed in our study. This model offers a novel tool that can assist clinicians in more accurately assessing patient risk and formulating personalized treatment plans. However, the study has certain limitations, such as the sample size and selection bias toward specific populations. Consistent with these findings, the ALBI score alone had suboptimal predictive power for ACLF in our study.

Our investigation revealed that CR levels differed significantly between the improvement group (median: 62.60 μmol·L^−1^, interquartile range [IQR]: 50.60, 73.80) and the mortality group (median: 79.10 μmol·L^−1^, IQR: 59.00, 115.00) in the training cohort, highlighting CR as an independent prognostic factor in ACLF patients, with higher levels predicting a worse prognosis. Elevated CR may indicate renal dysfunction, a hallmark of the multi-organ failure characteristic of ACLF, involving the kidneys, respiratory and circulatory systems (27–29). The increase in creatinine may not only indicate renal failure, but may also predict the deterioration of other organ functions, thereby influencing the overall clinical outcome (30–32). This finding is consistent with previous studies identifying CR as a marker of adverse prognosis in ACLF (33). In several prognostic models, CR is integrated with other clinical parameters to form complex predictive algorithms for ACLF (34–36). Notably, the ALBI score does not incorporate renal function metrics; therefore, we integrated CR with the ALBI score to potentially improve the predictive power of our model.

The role of Gender in ACLF is complex, involving enzyme activity, hormone levels, lifestyle factors and genetic predisposition (37). At the hormonal level, estrogen is known for its anti-fibrotic effects, which may contribute to the better prognosis observed in women with certain liver diseases (38–40). Lifestyle factors, such as alcohol consumption, are more prevalent in men, with excessive alcohol consumption directly damaging hepatocytes and indirectly accelerating the progression of liver failure through metabolic (41), inflammatory and nutritional effects. Genetically, recent evidence suggests that male-specific genes, such as the determinant region Y gene (42), act as potent profibrotic factors, which may explain the preponderance of males in our study (43). In conclusion, although gender plays a role in ACLF, it is typically not an independent prognostic factor and existing models often omit this variable. However, given the ease of assessing gender and the absence of this parameter in current models, we experimentally included it in our model. In the nomogram, women receive approximately 5 points, a relatively small contribution compared to other factors. We suggest that the complex interplay of multiple factors associated with gender may account for its suboptimal performance as an independent predictor of ACLF prognosis.

Ascites, a common complication of ACLF, typically results from portal hypertension (44), renal sodium and water retention, and decreased colloid osmotic pressure (45, 46). Although ascites is included in the Child-Pugh classification (47–49), our study simplifies its categorization as present or absent without assessing the volume of ascites and its impact on prognosis. Future research could include the quantification of ascites using ultrasound to refine prognostic assessments.

Our model, by assessing the 90-day prognosis of patients with ACLF, provides a robust decision-support tool for clinical practice. In clinical decision-making, by identifying those at high risk, physicians can formulate personalized treatment plans in advance, optimize resource allocation, and intervene promptly. In patient management, the application of this model can facilitate more precise resource allocation. By identifying patients who are most likely to benefit from specific treatments, the healthcare system can make more effective use of limited resources.

Due to the limitations of the study design and sample size, we included only a limited number of clinical variables. This may have constrained the model’s ability to comprehensively capture the complexity of patients’ conditions, thus affecting its robustness. From a model validation perspective, we stratified the 290 patients based on timeline to create a training set and a validation set, representing an internal validation process. This approach did not include data from patients in other hospitals for external validation. We hope that future research will involve collaborative multicentre studies to develop more accurate predictive models. The issue of gender in relation to ascites has been addressed previously and is therefore not discussed here. In terms of study design, our research is a retrospective cohort study. In order for the nomogram to be translated into clinical practice, we believe that further prospective clinical trials are essential to establish its clinical utility. Furthermore, the limited number of clinical variables included in our study may affect the robustness of the generated nomogram. Finally, our study lacks an aetiological classification for ACLF. The majority of our patients were infected with HBV, and although statistically categorizing the etiology and creating prognostic models for patients with different aetiologies should theoretically yield more accurate results, the current case numbers are insufficient for this purpose.

To improve the model’s predictive accuracy and clinical utility, we suggest that future research should consider incorporating a wider array of variables, including biomarkers, genetic markers, and lifestyle factors. Furthermore, we recommend conducting multicenter and multipopulation studies to verify the model’s universality and robustness. Concurrently, with the advancement of technology, we should explore the use of more advanced statistical and machine learning algorithms to optimize the model.

We suggest that in clinical application, the model could be integrated into electronic health record systems to provide clinicians with real-time prognostic assessments and treatment recommendations. In addition, the use of nomograms for patient education may improve patients’ understanding of their disease status and prognosis, thereby increasing their engagement in treatment. Finally, public health policy decisions based on the results of the model will provide data to support the prevention and control of liver disease.

In conclusion, the ALBI score combined with CR, gender and ascites to construct a survival prediction model not only improves the quality of clinical decision making, but also promotes the development of personalized and precise treatment and management of liver diseases, which has significant clinical importance and broad application prospects.

## Data Availability

The original contributions presented in the study are included in the article/supplementary material, further inquiries can be directed to WD, 18068111672@163.com.
